# Exploring the Use of Mobile and Wearable Technology among University Student Athletes in Lebanon: A Cross-Sectional Study

**DOI:** 10.3390/s21134472

**Published:** 2021-06-30

**Authors:** Marco Bardus, Cecile Borgi, Marwa El-Harakeh, Tarek Gherbal, Samer Kharroubi, Elie-Jacques Fares

**Affiliations:** 1 Department of Health Promotion and Community Health, Faculty of Health Sciences, American University of Beirut, Beirut 1107 2020, Lebanon; mb141@aub.edu.lb; 2 Department of Nutrition and Food Sciences, Faculty of Agricultural and Food Sciences, American University of Beirut, Beirut 1107 2020, Lebanon; ce37@aub.edu.lb (C.B.); md106@aub.edu.lb (M.E.-H.); sk157@aub.edu.lb (S.K.); 3 University Sports, Office of Student Affairs, American University of Beirut, Beirut 1107 2020, Lebanon; t.gherbal@amanhospital.org; 4 Aman Hospital, Doha, Qatar

**Keywords:** wearable electronic devices, athletes, students, fitness trackers, exercise

## Abstract

The markets of commercial wearables and health and fitness apps are constantly growing globally, especially among young adults and athletes, to track physical activity, energy expenditure and health. Despite their wide availability, evidence on use comes predominantly from the United States or Global North, with none targeting college student-athletes in low- and middle-income countries. This study was aimed to explore the use of these technologies among student-athletes at the American University of Beirut (AUB). We conducted a cross-sectional survey of 482 participants (average age 20 years) enrolled in 24 teams during Fall 2018; 230 students successfully completed the web-based survey, and 200 provided valid data. Fifty-three (26.5%) have owned a fitness tracker, mostly for self-monitoring. The most popular were Fitbit, Apple Watch, and Garmin. Similarly, 82 students (40%) used apps, primarily MyFitnessPal, Apple Health, and Samsung Health. Nevertheless, many participants discontinued use due to loss of interest or technical issues (breaking, usability, obsolescence, or lack of engagement). Wearable devices were considered superior to mobile phones alone as physical activity monitors. However, forming regular habits made self-monitoring via technology irrelevant. Further research is needed to better understand what motivates continuous use among student-athletes, who could use trackers to improve athletic performance and overall health.

## 1. Introduction

### 1.1. Wearables and Health Apps for Physical Activity Tracking

Wearable technology and smart clothing are gaining more and more attention in recent years. According to the industry analyst firm CCS Insight, wearable sales will reach $27 billion by 2022 [1]. In 2016, the consumer wearable market in the Middle East and North Africa region was on the rise, growing by 65% in one year [2]. In 2020, the regional market trend was predicted to reach 46 million users by 2022 [3]. Some recent reports show that the top four health and fitness apps are used chiefly for healthy eating, dieting and weight loss, exercise, and education on health [4], linked to wearable devices. Although the wearable market is still growing and can be considered a niche [5], there is a clear potential for the healthcare and fitness industries [6]. Wearable devices and fitness health apps are beneficial for research focusing on physical activity promotion and assessment [7]. Wearables could help assess various components of physical activity, such as: (1) total physical activity, (2) intensity, frequency, and duration of physical activity, (3) number of steps, strides, distance, and speed while walking, (4) classification of locomotive physical activities (walking, jogging, and running), (5) posture, (6) predicted total energy expenditure (TEE), predicted physical activity energy expenditure (PAEE), and predicted sleeping metabolic rate, (7) time of sleep and awake, and (8) heart rate and blood pressure [8].

Review evidence showed the potential for consumer wearable technologies to promote physical activity among the general public [9]. Wearable technologies are also used among athletes in elite sports to track their workouts and training sessions and evaluate their performance [10,11,12]. According to recent overviews of the literature, these technologies appear to be very useful for professional athletes [13], as they provide data to monitor training and enhance performance [14,15]. Commercial wearable devices seem to provide an accurate estimation of energy expenditure [16], can assist in increasing training load or weight management goals [17,18], and can be used to assess steps and heart rate in laboratory settings [19]. According to Seshadri et al. [11], the development of an all-encompassing device capable of measuring both biometric signs (stress, strain, and impact forces) and bio vital signs (glucose and lactic acid) is needed, and can serve as the next gold standard for this technology. These technologies seem to be well-accepted by most of the athletic body (athletes, coaches, sports scientists, exercise physiologists, and physiotherapists) due to their practicality, as they are lightweight and can be worn on different areas of the body (wrist, foot, waist, or hip); they can also assist patients in need of recovery [20]. The research opportunities in the area of wearable devices are limitless, and several reviews have analyzed the perspectives of specific populations, including overweight and obese adults [18], hospitalized adults [19], stroke survivors [20], healthy children and adolescents [21,22], or healthy adults [23]. However, evidence on the actual use of these devices in professional sports is still limited [24], especially in non-laboratory settings.

### 1.2. Wearables among Student Populations

Wearable technology is a helpful tool providing functional and social stimulation to individuals of all ages [25]. A few cross-sectional studies have investigated the use of wearable devices among the general population in Australia [26,27]. However, a growing evidence body shows that these technologies are appealing to young generations. For example, a U.S. national survey revealed that younger, healthier, and technologically literate adults were more likely to use these technologies [28]. Technology-savvy college students seem to have access to wearable technologies more than ever before [29,30]. A few studies have investigated the use of health and fitness apps among college students in the United States [31,32,33,34,35], finding overall support for their use, even if a randomized trial has shown null effects to encourage physical activity [35]. A systematic review shoed that wearable devices appeared to appeal to college students aged between 18 and 29 years old [36]. These years, defined as emerging adulthood, are a critical transitional period dictating future health behaviors [30,36]. Also qualitative evidence shows that college students are particularly interested in these technologies for activity tracking, with self-monitoring as the primary motivating factor [32]. Consistent with recent systematic review evidence, receiving concrete feedback allows youth to become more self-aware and hence, more encouraged and motivated to perform physical activity [36].

Similarly, mobile apps, which usually go together with wearable devices, have been found to embed persuasive features or behavior change techniques capable of triggering mechanisms of change in users [37]. A recent study found that students’ athletic identity was positively associated with ownership and usage of fitness trackers and apps [10]. This motivational readiness to change shows promise and provides an added value for technology use among young athletes in university settings [38]. However, to the best of our knowledge, there have been no previous studies assessing the use of digital technologies, including consumer wearable devices and health and fitness apps, among university student-athletes, and this evidence is particularly absent from low- and middle-income countries (LMICs), such as Lebanon.

### 1.3. Study Background

Lebanon is a small country in the East Mediterranean Region, which the World Bank considered an upper-middle-income country before being hit by compounded and unprecedented crises [39], which led to a decline of 19% of the GDP in 2020 and triple digit inflation [40]. In 2017, before the economic and financial crises hit the country, we formed a research team to consolidate the work of the American University of Beirut (AUB)’s University Sports in supporting the young athletes’ performance improvement. The team included faculty members from Health Sciences, Nutrition and Food Sciences. Motivated by studies assessing the use of health apps or wearable devices for tracking physical activity, diet, and weight management among the university population conducted by our research team’s behavioral and communication specialist [41,42], we wanted to expand this research to reach the student population. In January 2018, the physical therapist member of our research team observed that some students were adopting wearable devices and mobile phones during training sessions. This discovery prompted the team to conduct a study to systematically assess the readiness to use and reasons for actual use of wearable devices and health apps among the total population of AUB student-athletes.

### 1.4. Study Aims

The primary aim of this study was to explore and describe the current use of fitness trackers and health and fitness apps among AUB student-athletes and to study their motivation for using such technologies. The secondary aim of the study was to examine the associations between the use of fitness tracking devices with users’ characteristics and the use of health and fitness apps.

## 2. Materials and Methods

### 2.1. Study Design

This was a cross-sectional study based on an anonymous web-based survey, which was distributed via email among student-athletes enrolled at the American University of Beirut (AUB) in the Fall 2018 semester. We developed the questionnaire using LimeSurvey Version 3.14.8+180829 [43], which is AUB’s preferred web-based survey application, hosted on its servers. The research team members piloted the survey to test its usability and technical functionality before fielding the questionnaire. We report the methods according to the Checklist for Reporting Results of Internet E-Surveys (CHERRIES) [44].

The protocol of the study was approved by the Institutional Review Board (IRB) on 2 May 2018 (Ref. nr: SBS-2018-0138).

### 2.2. Setting

AUB is one of the oldest universities in the country, founded in 1866. AUB is a private, middle-sized university, ranked 220th in the QS World university rankings and second in the Arab region [45]. AUB is located in a 246,000 square meter campus overlooking the Mediterranean Sea that includes athletic fields, a private beach, a botanical garden, and 64 buildings hosting seven faculties: Agricultural and Food Sciences, Arts and Sciences, Engineering and Architecture, Health Sciences, Medicine, Nursing, and Business. The campus also comprises seven dormitories, five libraries, computer labs, a Medical Center, and a fitness center with a gym, pool, and outdoor sports facilities, including a football and rugby field, an athletic track, and tennis and basketball courts. The University Sports Department, which operates under the Office of Student Affairs, oversees the facilities and is responsible for training student-athletes competing in international and local sports events.

### 2.3. Participants and Procedures

We targeted student-athletes of any gender and age enrolled in the university in the fall 2018 semester. The total population of AUB student-athletes enrolled then was 482 (66% males, 34% females), with an average age of 20 (SD = 2, range: 17–46). They were enrolled in 24 different teams, distributed as follows (from the highest to the smallest proportion): male rugby (13%), male football (12%), male basketball (7%), female rugby, track and field (both genders), and badminton (6% each), swimming, and ultimate frisbee (5% each), female basketball, male futsal, archery, male handball, tennis, male water polo, and archery (4% each), and male volleyball, female volleyball, and table tennis (3% each).

We recruited a convenience sample of student-athletes as they were invited to participate in the study exclusively via email (we used a ‘closed survey’). The Office of Student Affairs provided us with a list of email addresses of all students enrolled in a sports club (i.e., the contact database), which we uploaded on LimeSurvey. The survey platform automatically generated 15-character long identifiers (so-called ‘tokens’) for each unique email address uploaded. The tokens were then appended to a survey URL, which was included in email invitations sent through LimeSurvey. Cookies were used to prevent repeated participation, and responses had the IP address logged. As we set the survey to be accessible only via tokens and to record anonymous responses, no data provided by participants could be linked back to the contact database, but the system allowed the blocking of repeated submissions. Access to the survey back-end was protected by a password and limited to two members of the research team (MB and EJF).

Potential participants received email invitations, including a survey URL. The first page of the survey included a consent form, which reminded the participants about the voluntary nature of the study. No incentives were offered. Once students agreed to participate in the study, they could proceed to the first page of the survey and answer the questions displayed. None of the questions were mandatory, and participants could freely navigate back and forth on each page of the survey. We sent up to three email reminders after the initial email invitation. Participants could complete the web-based questionnaire between 26 November 2018, and 5 December 2018.

### 2.4. Questionnaire

We developed a bespoke questionnaire for this study. The questionnaire consisted of seven pages or sections: (1) basic information, (2) use of wearable devices, (3) reasons for using wearable devices, (4), reasons for stopping using wearables, (5) use of health and fitness apps, (6) reasons for using such apps, and (7) reasons for stopping using apps.

The basic information page included questions aimed to describe the sample in terms of demographic information (gender; date of birth, used to calculate the exact age from the date of survey submission); anthropometrics (height and weight to calculate BMI; body fat and waist circumference, if known); and sports club membership.

The use of wearable devices page included questions about ownership of fitness trackers (i.e., “Have you ever owned a fitness tracker to track your activity or diet (e.g., Fitbit, Polar, Apple Watch)?”). The survey was set to include pipe logic questions, dynamically generated, based on the users’ responses. This allowed users to provide more detailed information about the brands owned (e.g., Fitbit, Polar, Garmin, Apple Watch) and whether participants were still using the device.

Respondents who declared using a device could then specify the reasons for using it among a set of multiple options (“I want to track my activity”, “I want to track my sleep”, “I want to track the water I drink”, “I want to track the calories I consume”, or “other reasons”, with an open-ended box), or to specify their reasons in an open-ended comment box.

Similarly, if respondents declared not to use a device anymore, they were asked why they stopped using it, with multiple options being: “It got broken”, “It got stolen”, “I lost interest in it”, “I was concerned about the device collecting my data” or “other reasons”. When selecting the latter option, participants could then further qualify their answers using an open-ended comment box.

Likewise, the use of digital technology page included questions about smartphone ownership (yes/no), operative system, and previous and current use of popular health and fitness apps to track activity or diet. Those who responded positively to this question were prompted to select among a list of popular apps in the “health and lifestyle” category (i.e., Apple Health, Samsung Health, Google Fit, MyFitnessPal, and Lifesum), or apps for “sports tracking” (i.e., Runtastic, Endomondo, Strava, Sports tracker, and Pacer) or “workout” apps (Freeletics, GetFit, and BetterMe). Participants could provide a name of alternative apps used. A follow-up question included the reasons for using the app (“I want to track my activity”, “I want to track my weight”, “I want to track my diet”, “I want to track the calories I consume”, or “other reasons”, with open-ended response).

If participants declared having stopped using an app, they were prompted to indicate the reasons for doing so. The list of reasons was based on the framework of the Mobile App Rating Scale (MARS) [46] and derived from previous research from a similar study conducted in the same institution [41]. The reasons were linked to the domains of engagement (“It was not engaging with me”; “I lost interest in it”), functionality (“It was not easy to use”), aesthetics (“It had too many annoying ads and pop-ups”), information (“I was concerned about my data”) and subjective quality (“It was expensive”; “It was of poor quality”) and other reasons (with open-ended response).

### 2.5. Analyses

Descriptive statistics were used to summarize the data. Correlations were used to assess any associations between technology use and demographic, anthropometric characteristics, and sport type. In addition to looking at a general pattern, we also checked for more specific patterns related to the studied major, conducting sub-group and sensitivity analyses using Chi-square tests (among categorical variables), ANOVA or t-tests (among nominal and continuous variables), as well as logistic regression to determine the association between usage of wearable technologies and sociodemographic factors. Due to the exploratory nature of the study, sample size calculations were not deemed necessary. The data were analyzed using the Statistical Package for Social Sciences (SPSSv24) for PC and Mac.

## 3. Results

### 3.1. Sample Characteristics 

**Figure 1:**
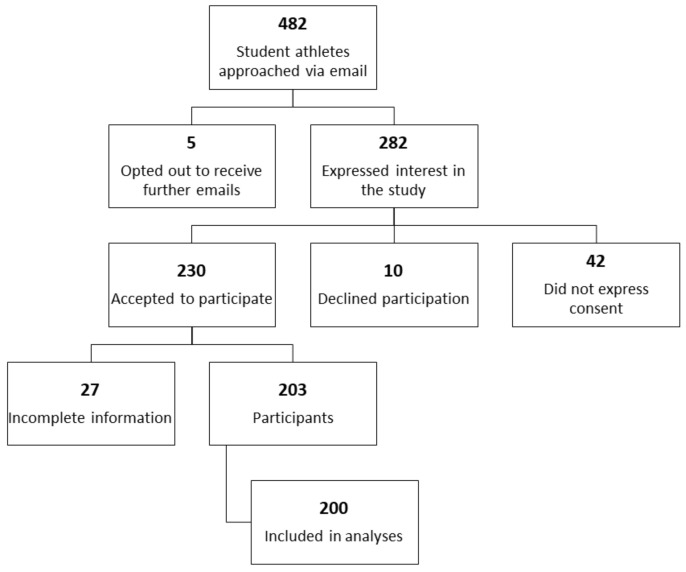
Participant recruitment flowchart.

Out of 482 student-athletes invited to participate in the study, 5 opted out to receive further email invitations (1.0%), and 282 showed some interest in the study by initiating the survey (view rate: 58.5%). Of these, 42 did not express consent (14.9%), 10 did not agree to participate in the study (3.5%), and 230 agreed to participate in the study (response rate: 81.5%; 47.7% of the total study population). Of those who agreed to participate in the study, 27 did not provide complete information and were excluded from the analyses (completion rate: 203/230, 88.3%). Out of the remaining 203 participants, 200 provided valid information on the use of fitness trackers, our primary outcome, and were included in the analyses (42% of the total population). The participant flow is provided in Figure 1 below. The characteristics of the analyzed sample are summarized in Table 1 below, which includes a breakdown of the sample by ever use of wearable technology. We did not include sample weights as we did not have demographic information on the student population.

Overall, the sample represents the entire AUB athlete population, as respondents were on average 20 years old (SD = 2.1, range 17–39), mostly males (65%), enrolled in rugby (21%) or football (13%) teams. Among those who reported anthropometric information useful to calculate the body mass index (*n* = 198), 65% were classified as being in the normal weight category (61% males, 39% females), and 25% as overweight (79% males, 21% females). There was no difference in age between male and female athletes, and the participants were almost equally distributed in teams, except for football (only males) and for track and field and swimming (mostly females). Males had significantly higher BMI (M_diff_ = 2.72, 95 CI = 1.68–3.77; t = 5.144, df = 196, *p* < 0.001), with females being more likely to be in the “normal” weight category compared to males (X^2^ (3, *n* = 198) = 26.440, *p* < 0.001, phi = 0.365). Males had also higher waist circumference (M_diff_ = 14.66, 95 CI = 6.63–22.69; t = 3.852, df = 17, *p* = 0.001) compared to females; whereas female athletes had higher percent body fat than males (M_diff_ = −7.04, 95 CI = −10.80–−3.28; t = −3.758, df = 53, *p* < 0.001). There were significant associations between ever users of wearable devices and sports team membership: those enrolled in the track and field team were more likely than others to have ever owned a device, whereas those enrolled in the ultimate frisbee team were less likely to do so.

There were no differences in technology use among male and female athletes, except for the operative system: males were less likely to own an iOS phone than females (X^2^ [1, *n* = 189] = 5.994, *p* = 0.014, phi = −0.190)—data not reported in the tables below.

### 3.2. Primary Objective 

#### 3.2.1. Ownership and Use of Wearable Devices and Fitness Apps

Out of 200 respondents, 53 (26.5%) declared having owned a wearable device to track their activity (34 males, 19 females), and 93 (46.7%) declared having used a health and fitness mobile app to track their activity or diet. Most of the athletes who had ever owned a wearable device were enrolled in the teams of rugby, track and field, and volleyball (9/53, 21%), followed by football, basketball, and water polo (6/53, 11%), see Table 1.

Table 2 summarizes the descriptive statistics related to the use of technology among the total sample and among athletes who ever used a fitness tracker device. The use of a fitness tracker was significantly associated with the operative system, with most users owning an iOS device (X^2^ [1, *n* = 194] = 15.057, *p* < 0.001, phi = 0.264), and with the use of health and fitness apps (X^2^ [1, *n* = 199] = 7.913, *p* = 0.006, phi = 0.199).

The most popular brands of wearable devices (Table 2) were: Fitbit (12% of the total cases), Apple Watch (9.5% of the total cases), and Garmin (4.0%). A small number of participants (*n* = 8) declared having owned multiple devices: 1 participant owned three devices (Fitbit, Polar, and Apple Watch), 7 participants owned two devices (6 owned a Fitbit and an Apple Watch; 1 a Fitbit and a Garmin device).

The most popular health apps were MyFitnessPal (21.5% of the total cases), Apple Health (10.0% of the cases), and Samsung health (5.5%). “Running” apps were popular among track and field disciplines, football, futsal, and swimming. “Workout” apps (i.e., Freeletics, GetFit); were less popular, and used among rugby, basketball, and handball players.

#### 3.2.2. Reasons for Using and Not Using Wearable Devices 

Nineteen out of 22 current wearable devices users reported their reasons (k) for using their device, which are summarized in Table 3. The most frequent reason was for tracking activity (57%), followed by sleep, calories consumed, and water intake. Four users further specified other reasons for using their devices, including monitoring performance, such as tracking heart rate (20-year old female rugby player, Apple Watch user), the pace (24-year old female track and field and tennis player, Garmin watch user), or speed while running (32-year old male water polo and track and field athlete, Polar user). Another reason included aesthetics: “It is a nice watch” (20-year old male water polo player, Samsung Gear Fit user).

Thirty-two out of the 54 owners of wearable devices explained their reasons for stopping using the devices (summarized in Table 3). In the majority of the cases, they lost interest in the device (57%), especially among Fitbit users (*n* = 11), followed by Apple Watch (*n* = 4), Misfit *(n* = 2), and Garmin (*n* = 1), or other brands (i.e., Mio, *n* = 1). The second most frequent reason for stopping using the device was that it got broken (24%); this was mostly the case of Fitbit (*n* = 4), Apple Watch (*n* = 3), Garmin (*n* = 1), and another brand (i.e., Nike Watch, *n* = 2). In two cases, the devices were stolen (one Apple Watch and one Fitbit), and in another case, one user lost their Misfit device.

Five users provided additional comments specifying their reasons for not using the devices. For example, one user provided a general comparative statement: “I thought Apple Watch was a better tracker” (20-year old female tennis player, Fitbit user). Two users mentioned technical faults, such as: “The battery fell during a game because it was raining so it must have slipped” (21-year old male football player, Fitbit user); “short battery life” (18-year-old, male football player, Apple Watch user).

Other reasons included lack of relevance for the athlete’s fitness goals, such as: “I didn’t think it was relevant to my physical activity anymore; I’m on the track team and do sprints mainly. No device really helps with timing short fast distances, and I’m already fit, (Also a nutrition student so I’m also very aware of my diet) and didn’t feel the need to log my food or water intake or how many steps I take per day. I regret buying it because it felt pretty useless to my case, and I used it only for telling the time–which I can simply check anywhere else. Perhaps it wasn’t the best suited device to get, but either way, I feel such devices are mainly for those trying to either lose weight or run long timed distances (a lot of the distance runner on the track team use Garmins)” (21-year old female track and field athlete, Fitbit user). “I wasn’t using it to track my fitness; it was just like any watch” (19-year old male basketball player, Apple Watch user).

When the survey was administered, among the 82 participants who declared having used a mobile app, 45 stated they were still using one (55%). Among the health and lifestyle apps chosen by the users, the most popular were included in the “health and lifestyle” category, including Apple Health (still used by 13/45, 29%), MyFitnessPal (10/45, 22%), Samsung Health (7/45, 16%), and Lifesum (1/45, 2%). The most popular “running” apps were Strava (4/45, 9%), Runtastic (3/45, 7%), Endomondo, and Pacer (both 1/45, 2%). Freeletics was the sole app in the workout category to be used. Other apps, such as the Nike Running Club and Garmin Connect, were still used by track and field athletes and rugby players.

#### 3.2.3. Reasons for Using Health and Fitness Apps

Forty-two out of 82 users of apps indicated their reasons for using an app, summarized in Table 4 below. Overall, the most frequent reason for using a “health and fitness” app was for tracking activity (64%), followed by tracking diet (17%), and weight (11%). Activity tracking was the preferred reason among users of “health and lifestyle” apps, such as Apple Health (*n* = 13), Samsung Health (*n* = 7), MyFitnessPal (*n* = 2), Lifesum (*n* = 1), Huawei Health (*n* = 2), and Google Fit (*n* = 1). Activity tracking was the preferred reason for using “running apps”, such as Nike Running Club (*n* = 4), Runtastic (*n* = 3), Strava, Endomondo Pacer, and Garmin Connect (all with *n* = 1). MyFitnessPal was the most used app both for weight tracking and for dietary tracking (*n* = 5).

Among other reasons, an 18-year old male football player, a user of Freeletics, stated he used the app for “staying fit”. A 21-year old female rugby player used Lifesum to keep track of her water intake. A 20-year old male futsal player, Apple Health user, added that he used to app “Just to check my average walking distance per day”. Two female Nike Running Club users (a 19-year old swimmer and a 20-year old handball player) used the app to “track my runs” and “track my running pace”, respectively.

Fifty of the 82 app users (61%) explained their reasons for stopping using the apps (see Table 4). In the majority of the cases, users lost interest in the app (56%) or found the app not engaging (16%), not easy to use, and with too many ads and pop-ups (9%). Losing interest in the app was the main reason for discontinuing MyFitnessPal use (*n* = 26), an app that was also deemed not easy to use (*n* = 6) and not engaging (*n* = 5).

Some users provided comments to explain additional reasons for quitting the use of apps. Two users mentioned they stopped using the apps as they became impractical: “I didn’t want to hold my phone while I run” (21-year old, male track & field athlete, Strava user); “My wearable Polar device better fits my needs” (32-year old male water polo and track and field athlete, Endomondo user).

MyFitnessPal users indicated the difficulty and inaccuracy of logging food: “[It] doesn’t track calories lost as well as a Fitbit” (21 year-old female, archery athlete); “Tracking day-to-day calories was relatively hard as some menu items weren’t on the app. I felt that some of the values weren’t accurate” (male, rugby player). Other reasons included reaching the desired goals or changing the priorities due to external events, as expressed in the following comments: “I lost the weight I desired to lose” (19-year-old male basketball player). “I developed a workout routine, and no longer felt the need of having such an app” (19-year-old, male Rugby player); “I stopped using it because I was injured and was no longer concerned with my diet” (21-year-old male futsal player). Another Runtastic user said: “I only used in when running outside of the field, and I don’t do that anymore” (21-year-old male rugby player).

### 3.3. Secondary Objective

#### Factors Associated with the Use of Wearable Devices

We explored the associations between socio-demographic, health characteristics, and technology utilization of health and fitness tracking devices (see Table 5). We found that age was positively and significantly associated with the use of these devices (adjusted OR: 1.25, 95% CI: 1.041, 1.50, *p* = 0.018). Holding constant gender, sports category, health profile variables, and the use of mobile apps, with every increase in years, the odds of owning a wearable increased by 25%. Additionally, participants who reported having used health and fitness apps had significantly higher odds of using fitness tracking devices (Adjusted OR: 2.61, 95% CI: 1.32, 2.18, *p* = 0.006). This means that holding constant age, gender, sports category, and health profile variables, the odds of owning a fitness tracker were 161% higher among those who used health and fitness apps. No other significant associations were detected.

## 4. Discussion

### 4.1. Use of Fitness Trackers and Health Apps among University Student Athletes

The primary aim of this study was to explore and describe the use of fitness trackers and health and fitness apps among university student-athletes enrolled at the American University of Beirut (AUB), Lebanon in the Fall 2018 semester. Our findings show that about a third of our sample (53/200, 27%), representing about 41% of the entire population of AUB student-athletes, had owned a wearable device. Our figures align with the proportion of participants who declared using wearable devices in similar cross-sectional studies, which targeted college students in the United States [31,32,33,34,35]. Notably, this segment of the population represents the ‘innovators’ or ‘early adopters’ of technology in the niche market of wearable devices. However, this study can have implications for manufacturers and can assist in product development for wearable users, who might share the same opinions and usage patterns.

#### 4.1.1. Reasons for Using Wearable Devices

In our study, wearable devices were used to set goals and self-monitor behaviors, such as physical activity, but also sleep, calories eaten and consumed, and water consumption; “monitor sleep” and “losing weight” were among the preferred reasons reported in Kinney et al.’s study [31] and activity tracking in Papalia and colleagues’ [34]. Goal setting and self-monitoring are the most valuable features of wearable devices as reported in qualitative studies [32] and content analyses of mobile apps for physical activity promotion and weight management [37].

Our data shows that most wearable fitness tracker owners eventually stopped using them after initially using them. Most of the sample (57%) stopped using their devices due to lost interest, similarly to what was reported in a US study (i.e., 50%) [31]. Another reason for discontinuation of use was that the device broke down (24%), which was also noted in an Australian cross-sectional study among the general population (22%) [27]. A potential explanation for this phenomenon might be due to the brands of devices used. In our study, Fitbit (44%) and Apple Watch (35%) were the most popular brands, similar to what was reported in two studies [31,34]. The proportion of Apple Watch users in our study was aligned with market trends, whereas Fitbit users were an anomaly, as the global market share of this brand was around 5% in 2019, losing more than 30% from 2014 [47]. Fitbit is known for revamping their product line every six months or less, with the average duration of a product between 1 and 2 years, due to malfunctions. [48].

#### 4.1.2. Reasons for Using Health and Fitness Apps

When it comes to health and fitness apps, 82 out of 200 (40%) student athletes declared having used an app to track their activity or diet. The majority of our study participants used apps to track their activity (64%), followed by diet (17%), and weight (11%). Similar reasons were reported among college students in similar studies [32,49] and are aligned with national trends among the U.S. general population [50]. In this context, we found the most popular apps to be in the “health and lifestyle” category. This finding is consistent with the literature about the use of health apps among the general public, where diet, weight, and physical activity were the primary drivers for downloading apps [37,50,51].

Similar to the wearables, participants discontinued using health and fitness apps as they were not interesting (56%), not engaging enough (16%), not easy to use, or due to too many ads and pop-ups (9%), or they were pricy (2.7%). These findings are aligned with a U.S. national survey, in which about half of the sample (45.7%) had stopped using the apps after some time [51]. They are also consistent with some qualitative evidence showing that users stop using health apps due to hidden costs, increased data entry burden [52], and low engagement [53]. Discontinuing app usage was also reported in a user-centered study conducted among employees of the same institution [41], in which the authors found that mobile apps are not used if not engaging and easy to use.

The health app market is highly volatile and unstable; some reports showed that the average app turnover was 3.7 days in Google Play (for Android phones) and 13.7 days in iTunes (for iOS phones) over nine months [54]. Other research shows that many apps are downloaded less than 500 times or never used [55]. This instability and unpredictability of the health app market pose several challenges for developers and researchers. The problem of engagement with wearable and mobile technologies has been previously reported and discussed in the literature [37,56,57]. Patel and colleagues [3] argued that the successful, continued use of fitness devices depends primarily on engagement strategies, which prompt human behavior, focusing on encouragement, social competition and collaboration, and feedback loops. For example, the sustained utilization of mobile apps among U.S. college students was associated with features, such as recordability, networkability, credibility, comprehensibility, and trendiness of the apps [58]. App developers, wearable designers, and makers need to address engagement and make their products more durable.

There might be other reasons for stopping using health and fitness trackers and apps. One explanation is that the device and self-monitoring tool becomes obsolete when the promoted behavior becomes a routine or a habit. Some evidence on habit formation in the physical activity domain [59] suggests that although self-monitoring is essential for the initiation and maintenance of healthy behaviors, some people eventually learn how to self-motivate without the help of external cues, including technology [26]. In this study, some users declared to stop using the devices and activity trackers once physical activity became a habit, but this is contrary to some studies investigating the use of wearable technology among older adults [60]. However, this remains speculation due to the cross-sectional nature of our study. Longitudinal, experimental studies are needed to confirm this hypothesis.

### 4.2. Use of Wearable Devices and Sociodemographic Factors

The study’s second objective was to examine the associations between users’ characteristics and the use of tracking devices. Our analyses showed a significant association between age and the use of mobile apps. We did not find significant associations between wearable use and other sociodemographic factors, or health profile, including BMI, consistent with findings from previous studies focusing on wearable use among college students [31,33,34]. In our study, younger students who used a health and fitness app were significantly less likely to own a wearable device compared to their older counterparts, who had not used an app. While the entire sample was quite young (average 20 years) and age was not different in the sub-sample of wearable users and non-users, age and use of health and fitness apps were both significantly associated with owning a wearable device. This finding is unique to this study, as age was not significantly associated with wearable use in Kinney et al.’s study [31], which recruited 356 students with a broader age spectrum (range 18–29), nor reported as a factor in Blackstone and Herrmann’s [33], nor in Papalia et al.’s [34] studies. The relationship between mobile and wearable technologies was also reported in the previously cited US-based study among college students, where 84% of the sample used their fitness tracker’s corresponding mobile health app [31]. This finding is not surprising, as most wearable devices have a companion mobile app, which allows users to change device settings, or utilize additional features via Bluetooth, Wi-fi connections, or proprietary dongles.

This study provided information about the proportions of users of these technologies among the student-athlete population at AUB. It offers a unique insight into their attitudes and preferences, laying the ground for other research studies and interventions. The more we understand this specific population, the better we can address its needs and improve its athletic training and performance using technology. However, usage trends warrant further exploration. This survey can be used as a baseline for future cohort studies monitoring the use of these technologies in our university and in other academic institutions in Lebanon and in the whole region. We suggest including our brief questionnaire in the enrolment phase of student-athletes at the beginning of each academic year in order to assess any changes in technology adoption.

### 4.3. Strengths and Limitations

To the best of our knowledge, this is the first study that examined the use of wearable devices and related health apps among student-athletes and the first study of this kind to be conducted in Lebanon. Even though several cross-sectional studies on the use of wearable devices and mobile apps among college students have been reported in the literature, these were predominantly from the United States; no evidence appeared so far from the Middle East, whether from high- or low-income countries. Some limitations need to be acknowledged. First, our data were collected cross-sectionally, while participants’ use of wearable devices and health apps may have evolved, as technology is continuously evolving. Second, the small sample size does not allow the generalization of results beyond the study population. Although our sample is representative the student-athlete population at AUB, and although our findings align with other similar studies conducted in other parts of the world, generalizations cannot be made due to the nature of this kind of research. Some self-selection bias might be due to the recruitment, which was based on convenience sampling techniques; we invited all potential student-athletes, but only those interested, and who agreed to participate in the study could complete the questionnaire. Additionally, we need to acknowledge the potential of reporting bias, as the survey was self-administered. In other words, we can conclude that we have suggestive evidence indicating that student-athletes in a university campus in the Middle East show usage patterns compared to similar populations in the United States and Australia. Larger, nationally representative, and cross-country studies should be conducted to evaluate the status of wearable use among student-athletes. Longitudinal, cohort studies are required to study how student-athletes use wearable devices, and what motivates their continuous use.

## 5. Conclusions

While some student-athletes use wearable devices and health apps to track their activity and monitor their health behaviors, these devices are used by a relatively small number of people, which constitutes a niche market. Notably, many student-athletes stopped using apps and wearables as they lost interest in them, or due to technical faults of the devices themselves. Longitudinal studies are needed to understand how student-athletes use these technologies in their daily routines and what could prompt continued use so that these devices could be effectively used to improve student athletes’ fitness and performance.

## Figures and Tables

**Table 1:** Sample characteristics for the total sample among ever users and non-users of wearable devices ^†^.

Participants’ Characteristics	Sample (n = 200)	Non-Users (n = 147)	Users (n = 53)	*p*-Value ^‡^
*Demographics*				
Age (years) ^a^	20.22 ± 0.16	19.97 ± 0.11	20.87 ± 0.48	0.075
Gender ^b^				
Females	68 (34.7)	49 (34.3)	19 (35.8)	0.836
Males	128 (65.3)	94 (65.7)	34 (64.2)	
Sport team ^c^				
Rugby	42 (21.2)	33 (22.8)	9 (17.0)	0.379
Football	26 (13.1)	20 (13.8)	6 (11.3)	0.648
Ultimate frisbee	18 (9.1)	17 (11.7)	1 (1.9)	0.047
Track & Field	16 (8.1)	7 (4.8)	9 (17.0)	0.009
Swimming	16 (8.1)	12 (8.3)	4 (7.5)	1.000
Handball	16 (8.1)	11 (7.6)	5 (9.4)	0.769
Basketball	16 (8.1)	10 (6.9)	6 (11.3)	0.377
Futsal	11 (5.6)	6 (4.1)	5 (9.4)	0.168
Water polo	12 (6.1)	6 (4.1)	6 (11.3)	0.088
Volleyball	11 (5.6)	2 (3.8)	9 (6.2)	0.731
Badminton	9 (4.5)	8 (5.5)	1 (1.9)	0.449
Table tennis	7 (3.5)	4 (2.8)	3 (5.7)	0.387
Tennis	6 (3.0)	4 (2.8)	2 (3.8)	1.000
Archery	6 (3.0)	5 (3.4)	1 (1.9)	0.686
*Sport Category*				
Individual	51 (25.8)	36 (24.8)	15 (28.3)	0.621
Individual and group	147 (74.2)	109 (75.2)	38 (71.7)	
*Health profile*				
BMI(kg/m^2^) ^d^	23.89 ± 0.27	23.66 ± 0.30	24.52 ± 0.56	0.157
BMI category ^d^				
Underweight/Normal	137 (69.2)	104 (71.7)	33 (62.3)	0.202
Overweight/Obese	61 (30.8)	41 (28.3)	20 (37.7)	
Body fat mass (%) ^e^	16.73 ± 0.87	17.20 ± 1.02	15.65 ± 1.67	0.414
Underfat	13 (24.1)	7 (18.9)	6 (35.3)	
Standard Minus/Plus	34 (63.0)	4 (64.9)	10 (58.8)	
Overfat/Obese	7 (13.0)	6 (16.2)	1 (5.9)	
Waist circumference (cm) ^f^	77.85 ± 2.37	77.50 ± 3.38	78.20 ± 3.51	0.887

^†^ Continuous variables were presented as means and standard errors (SE), whereas categorical variables were reported as frequencies (n) and proportions (%). ^‡^ Independent and chi-square *t*-tests were conducted to assess differences in sample characteristics among users and non-users of health and fitness wearable devices. ^a^ n = 190, as 10 participants did not provide a valid date of birth; ^b^ n = 196, as 4 participants did not provide gender information; ^c^ n = 198, as 2 participants did not indicate a sports team; ^d^ n = 198, as 2 participants did not provide valid weight information; ^e^ n = 56, as 144 participants did not provide valid body fat information; ^f^ n = 20, as 180 participants did not provide valid waist circumference information.

**Table 2:** Use of technology among the total sample, and among current users and non-users of health and fitness wearable devices.

	Total Sample (n = 200)	Current Non-Users (n = 147)	Current Users (n = 53)	*p*-Value ^‡^
*Phone ownership*				0.733
Yes	194 (97.0)	142 (96.5)	52 (98.1)	
No	1 (0.5)	1 (0.7)	0 (0.0)	
Missing/Don’t know	5 (2.5)	4 (2.7)	1 (1.9)	
*Phone Operative System*				<0.001
Android	57(29.4)	52 (35.4)	5 (9.4)	
iOS	134(69.1)	88 (59.9)	46 (86.8)	
Both	3(1.5)	2 (1.4)	1 (1.9)	
Missing/Don’t know	6 (3.0)	5 (3.4)	1 (1.9)	
*Brand of ever owned wearable tracking device*				
Fitbit	24 (12.0)	19 (12.9)	3 (5.7)	1.000
Apple watch	19 (9.5)	9 (6.1)	10 (18.9)	1.000
Garmin	8 (4.0)	3 (2.0)	5 (9.4)	1.000
Polar	3 (1.5)	0 (0.0)	3 (5.7)	1.000
Misfit	2 (1.0)	0 (0.0)	2 (3.8)	1.000
Samsung gear fit	1 (0.5)	0 (0.0)	1 (1.9)	1.000
Other	5 (2.5)	1 (0.7)	4 (7.5)	1.000
Missing/Don’t know	147 (73.5)	-	-	
*Ever used a health and fitness app*				0.006
Yes	93 (46.5)	60 (40.8)	33 (62.3)	
No	106 (53.0)	87 (59.2)	19 (35.8)	
Missing/Don’t know	1 (0.5)	0 (0.0)	1 (1.9)	
*Currently using a health and fitness app*				0.673
Yes	45 (22.5)	30 (20.4)	15 (78.9)	
No	50 (25.0)	31 (21.1)	19 (35.8)	
Missing/Don’t know	105 (52.5)	86 (58.5)	19 (35.8)	
*Brand of ever used health and fitness app*				
My Fitness Pal	43 (21.5)	27 (18.4)	16 (36.1)	0.829
Apple Health Kit	20 (10.0)	9 (6.1)	11 (20.8)	0.063
Samsung Health	11 (5.5)	10 (6.8)	1 (1.9)	0.090
Runtastic	9 (4.5)	7 (4.8)	2 (3.8)	0.484
Strava	5 (2.5)	1 (0.7)	4 (7.5)	0.052
Freeletics	4 (2.0)	3 (2.0)	1 (1.9)	1.000
SportsTracker	3 (1.5)	1 (0.7)	2 (3.8)	0.550
Lifesum	3 (1.5)	3 (2.0)	0 (0.0)	0.306
GetFit	3 (1.5)	2 (2.0)	1 (1.9)	1.000
Endomondo	2 (1.0)	1 (0.7)	1 (1.9)	1.000
Pacer	1 (0.5)	0 (0.0)	1 (1.9)	1.000
Other	9 (4.5)	7 (4.8)	2 (3.8)	0.484
Missing/Don’t know	118 (59.0)	94 (63.9)	24 (45.3)	

^‡^ Chi-square *t*-tests were conducted to assess differences in sample characteristics among users and non-users of health and fitness wearable devices.

**Table 3:** Reasons for using and not using wearable devices.

Reasons	Responses *k* (%)	Brands Mentioned (Number of Users)
*Reason for using* (*k* = 37)		
I want to track my activity	21 (56.8)	Fitbit (3), Polar (2), Garmin (5), Apple Watch (9), Suunto (1), Mi Fit 2 (1)
I want to track my sleep	6 (16.2)	Fitbit (2), Polar (1), Garmin (1), Apple Watch (2)
I want to track the calories I consume	5 (13.5)	Fitbit (1), Polar (2), Apple Watch (2)
I want to track the water I drink	1 (2.7)	Fitbit (1)
Other reasons	4 (10.8)	Apple Watch (1), Polar (1), Garmin (1), Samsung Gear (1)
*Reason for not using* (*k* = 42)		
I lost interest in it	24 (57.1)	Fitbit (11), Garmin (1), Apple Watch (4), Misfit (2), Mio (1),
It got broken	10 (23.8)	Fitbit (4), Garmin (1), Apple Watch (3), Nike Watch (2)
It got stolen	3 (7.1)	Fitbit (1), Apple Watch (3)
Other reasons	4 (9.5)	Fitbit (3), Apple Watch (2), Misfit (1)

**Table 4:** Reasons for using and not using health and fitness apps.

Reasons	Responses *k* (%)	Brands Mentioned (Number of Users)
*Reason for using* (*k* = 64)		
I want to track my activity	41 (64.1)	MyFitnessPal (2), Strava (1), Endomondo (1), Runtastic (3), Pacer (1), Apple Health (13), Samsung Health (7), Nike Running Club (4), Huawei Health (2), Google Fit (1), Garmin Connect (1), Unspecified (3)
I want to track my weight	7 (10.9)	MyFitnessPal (5), Lifesum (1), Apple Health (1)
I want to track my diet	11 (17.2)	MyFitnessPal (5), Strava (1), Lifesum (1), Apple Health (1), Nike Running Club (1),
Other reasons	5 (7.8)	Freeletics (1), Lifesum (1), Apple Health (1), Nike Running Club (2)
*Reason for not using* (*k* = 110)		
I lost interest in it	61 (55.5)	MyFitnessPal (26), SportsTracker (1), Freeletics (3), Runtastic (3), Lifesum (2), GetFit (2), Apple Health (4), Samsung Health (4), Unspecified (17)
It was not engaging with me	17 (15.5)	MyFitnessPal (5), SportsTracker (1), Runtastic (2), Lifesum (1), GetFit (1), Apple Health (3), Samsung Health (1), Unspecified (3)
It was not easy to use	10 (9.1)	MyFitnessPal (6), SportsTracker (1), Runtastic (1), GetFit (1), Samsung Health (1)
It had too many annoying ads and pop-ups	10 (9.1)	MyFitnessPal (4), Freeletics (1), Runtastic (1), Lifesum (1), GetFit (1), Apple Health (1), Unspecified (1)
It was too expensive	3 (2.7)	Freeletics (1), Runtastic (1), Unspecified (1)
It was of poor quality	1 (0.9)	Runtastic (1)
Other reasons	8 (7.2)	MyFitnessPal (4), Strava (1), Endomondo (1), Runtastic (1)

**Table 5:** Associations of socio-demographic, health characteristics and technology use with the use of health and fitness tracking devices.

Participants’ Characteristics	Have You Ever Owned a Fitness Tracker to Track Your Activity or Diet (e.g., Fitbit, Polar, Apple Watch)?	
	Unadjusted Odds Ratio (95% CI)	Adjusted Odds Ratio (95% CI)
*Demographics*		
Age (years)	1.20 (1.02, 1.44), *p* = 0.029	1.25 (1.04, 1.50), *p* = 0.018
Gender		
Females	1.0	
Males	0.93 (0.48, 1.80), *p* = 0.836	-
Sport category		
Individual sport	1.0	
Group sport	0.77 (0.38, 1.59), *p* = 0.490	-
Individual and group sport	2.40 (0.53, 10.88), *p* = 0.256	-
*Health profile*		
BMI category		
Underweight/Normal	1.0	
Overweight	1.62 (0.80, 3.28), *p* = 0.177	-
Obese	1.18 (0.30, 4.71), *p* = 0.813	-
Body fat mass	0.96 (0.88, 1.05), *p* = 0.408	-
Waist circumference (cm)	1.01 (0.92, 1.09), *p*-0.880	-
*Use of technology*		
Ever used health and fitness mobile apps	2.52 (1.31, 4.84), *p* = 0.006	2.61 (1.32, 2.18), *p* = 0.006
Currently using a mobile app	0.82 (0.35, 1.89), *p* = 0.636	-

## Data Availability

The data presented in this study are openly available from the American University of Beirut’s repository AUB Scholar Works. This data can be found here: http://hdl.handle.net/10938/22911 (accessed on 14 June 2021).
